# Charting the Uncharted: Clinico-Microbiological Profile of Bacteria Isolated From Bronchoalveolar Lavage Samples of Patients With Lower Respiratory Tract Infections at a Tertiary Care Hospital in Odisha, India

**DOI:** 10.7759/cureus.106731

**Published:** 2026-04-09

**Authors:** Rumita Dey, Kunalsen Jagatdeo, Kumudini Panigrahi, Basanti Kumari Pathi

**Affiliations:** 1 Microbiology, Kalinga Institute of Medical Sciences, Bhubaneswar, IND

**Keywords:** antimicrobial susceptibility test, bronchoalveolar lavage, enterobacterales, klebsiella, lrti

## Abstract

Background and objectives

Lower respiratory tract infections (LRTIs) are a major cause of morbidity and mortality worldwide, with growing diagnostic challenges due to evolving bacterial profiles and antimicrobial resistance. This study aimed to evaluate the clinico-microbiological spectrum of bacteria isolated from bronchoalveolar lavage (BAL) samples in patients with suspected LRTIs and to analyze their antimicrobial susceptibility patterns at a tertiary care hospital in Odisha, India.

Methods

A retrospective observational study was carried out over three years, during which BAL samples from adult hospitalized patients with clinically diagnosed LRTIs were processed using established microbiological techniques. Bacterial identification and antimicrobial susceptibility testing were performed following standard microbiological procedures. Demographic data and comorbidities were also evaluated.

Results

Of 390 BAL samples, 222 (56.9%) yielded significant pathogenic growth. A male predominance and a high prevalence of comorbidities, particularly diabetes mellitus, were noted among the patients. Gram-negative bacteria constituted the majority of isolates, with *Klebsiella pneumoniae* as the most frequently isolated pathogen. Gram-negative isolates showed a high rate of resistance to β-lactams, cephalosporins, and fluoroquinolones, while Gram-positive cocci remained uniformly susceptible to vancomycin and linezolid.

Conclusions

The study highlights a predominance of Gram-negative pathogens in LRTIs and confirms BAL as an effective diagnostic modality. Continuous regional surveillance and prospective multicentric studies incorporating molecular resistance profiling are essential to guide rational empirical therapy and strengthen antimicrobial stewardship.

## Introduction

Lower respiratory tract infections (LRTIs) are among the most common causes implicated in causing significant mortality and morbidity around the world. They usually present with symptoms like severe cough, dyspnoea, production of sputum, and wheezing, all of which may be accompanied by discomfort or pain in the thoracic region. Acute cases generally last one to three weeks [1]. Untreated or unresolved LRTIs are a leading cause of mortality from infectious diseases in both low-income and high-income countries [2]. They remain a major public health concern across all age groups, particularly among young children and the elderly [3].

A major concern with LRTIs is misdiagnosis due to the variability and frequent non-specificity of the presenting symptoms. The repertoire of implicated bacteria is also expanding and evolving [4]. Chronic respiratory diseases like chronic obstructive pulmonary disease (COPD), cystic fibrosis, occupational lung diseases, and asthma often worsen with infections and their sequelae, leading to an often irreversible decline in lung function [5,6]. One of the major contributors to the alarming levels of mortality seen in LRTIs is the lack of timely availability of organism identification and antimicrobial susceptibility profiles. Efficiency in the isolation of the causative agent depends largely on the appropriateness and quality of the sample sent for diagnosis.

Bronchoalveolar lavage (BAL) samples are now considered the gold standard for the diagnosis of LRTIs [7]. Sputum samples show a very low diagnostic yield of 24% due to frequent contamination with oral flora [8], whereas BAL samples overcome this limitation and significantly reduce such contamination, allowing for more specific pathogen isolation [9]. Another major factor that contributes to the high rates of adverse outcomes of LRTIs is the inappropriate use of antibiotics before culture and antimicrobial susceptibility results become available [10].

Empiric therapy needs to be chosen carefully and prescribed with the required dosage in order to obtain the best therapeutic response, minimize adverse effects, and prevent the emergence of resistance in the pathogen [11]. However, the treatment choice is often based on the apparent antimicrobial spectrum of the drug. If an in-house antibiogram is not regularly updated and consulted while prescribing empirical therapy, any resistance that the pathogen might harbor can compromise the treatment course for the patient [12]. These kinds of choices may lead not only to therapy failure but also contribute to the establishment of resistant strains of the organism. This may further lead to increased mortality, morbidity, and healthcare costs for both the affected patients and others through nosocomial infections [13-14].

There is a growing need for widespread research in our country on the use of BAL for the early isolation of pathogens involved in LRTIs. This study was undertaken to establish the microbiological profile of BAL fluid in patients with suspected lower respiratory tract infections and to evaluate antimicrobial susceptibility patterns to guide effective treatment strategies in a tertiary care hospital setting in Odisha.

## Materials and methods

Study design, setting, and ethical approval

This retrospective was conducted from May 2022 to May 2025 at the Department of Microbiology, Kalinga Institute of Medical Science, Bhubaneswar, after the approval of the institutional IEC (KIIT/KIMS/IEC/2304/2025, dated 22-09-2025). This study adheres to the reporting of observational studies in Epidemiology (STROBE) guidelines.

Eligibility criteria

Inclusion Criteria

Inclusion criteria were all the culture reports of BAL samples received from adult patients admitted during the study period.

Exclusion Criteria

Exclusion criteria were BAL samples received from the pediatric age group and those with incomplete and missing reports.

Sample processing

Standard procedures were used to collect the BAL sample, transport, and process it for any microbial growth identification and antimicrobial susceptibility testing.

Specimen collection

As per standard collection procedures routinely followed in the institution, around 30-50 ml of normal saline was infused into the lung using a fibrotic bronchoscope connected to peripheral bronchiolar branches and aspirated immediately. The fluid was collected in a sterile, dry, leakproof, screw-capped container and transported to the laboratory along with demographic data of the recruited participants, including age and gender, while observing proper precautions. The BAL sample was processed in the laboratory without delay. In cases where delay was unavoidable, the samples were kept at room temperature. Smears were prepared from the sample using a 2 mm sterile loop and stained with Gram’s stain.

The samples were inoculated in 5% sheep blood agar, MacConkey agar, and chocolate agar and incubated overnight in a 5% CO_2_ incubator. The plates were read once after 24 hours and again after 48 hours. Plates having a growth of >10^4^ CFU/ml were subjected to further identification processes as follows. The bacterial colonies grown were again subjected to Gram`s staining to classify them as Gram-positive or Gram-negative bacteria. Identification and antimicrobial susceptibility testing by following standard and CLSI (2022 to 2025)guidelines using the VITEK 2 system (bioMérieux, Marcy l'Etoile, France) [15-18].

Statistical analysis

This statistical analysis was conducted using R software (version 4.4.3; R Development Core Team, Vienna, Austria) for data computation [19].

## Results

During the study period, 390 BAL samples were analyzed according to the inclusion criteria from adult patients with suspected LRTIs. Of these, 222 samples showed growth of a pathogenic organism, yielding a culture positivity rate of 56.9%. Further demographic analysis was performed on the samples with positive growth. These 222 patients had a mean age of 53.9 years. Notably, 166 (74.8%) were males, and 56 (25.2%) were females. Significantly, 157 (70.7%) reported a history of type 2 diabetes mellitus, and 81 (36.5%) were reported to have hypertension, as shown in Table 1.

**Table 1:** Demographic distribution SD: standard deviation

Parameters	Value
Total participants with positive cultures, n	222
Age, years, mean ± SD	53.9 ± 8.3
Sex, n (%)	
Female	56 (25.2%)
Male	166 (74.8%)
Comorbidities, n (%)	
Type 2 diabetes mellitus	157 (70.7%)
Hypertension	81 (36.5%)

Of note, 122 (55%) of the 222 isolates belonged to Enterobacterales, of which the most prolifically isolated organism was Klebsiella pneumoniae (84/222, 37.8%). Other Enterobacterales isolates included Escherichia coli (18/222, 8.1%), Enterobacter cloacae complex (12/222, 5.4%), Proteus mirabilis (4/222, 1.8%), Morganella morgagnii (2/222, 0.9%), and Providentia stuartii (2/222, 0.9%). Non-fermenters formed 35.1% (78/222), including Pseudomonas aeruginosa (43/222, 19.4%), Acinetobacter baumannii (30/222, 13.5%), Burkholderia cepacia (3/222, 1.4%), and Elizabethkingia meningoseptica (2/222, 0.9%). Gram-positive cocci were found to make-up 9.9% (22/222) of the pathogens isolated, including Staphylococcus aureus (9/222, 4.1%), Streptococcus pneumoniae (7/222, 3.2%), Enterococcus faecalis (3/222, 1.4%), and Enterococcus faecium (3/222, 1.4%) (Table 2).

**Table 2:** Distribution of the pathogens isolated from positive BAL culture BAL: bronchoalveolar lavage

Pathogens	N (%)
Bacterial isolates	
Enterobacterales	122 (55.0%)
Escherichia coli	18 (8.1%)
Klebsiella pneumoniae	84 (37.8%)
Enterobacter cloacae complex	12 (5.4%)
Proteus mirabilis	4 (1.8%)
Morganella morgagnii	2 (0.9%)
Providentia stuartii	2 (0.9%)
Non-fermenters	78 (35.1%)
Acinetobacter baumannii	30 (13.5%)
Pseudomonas aeruginosa	43 (19.4%)
Burkholderia cepacia	3 (1.4%)
Elizabethkingia meningoseptica	2 (0.9%)
Gram-positive cocci	22 (9.9%)
Staphylococcus aureus	9 (4.1%)
Enterococcus faecalis	3 (1.4%)
Enterococcus faecium	3 (1.4%)
Streptococcus pneumoniae	7 (3.2%)

Antimicrobial susceptibility tests conducted showed a variable and interesting pattern among the isolated pathogens, as depicted in Tables 3-5.

**Table 3:** Antimicrobial susceptibility test findings of Enterobacterales (n = 122) AMC: amoxicillin-clavulanic acid; TZP: piperacillin-tazobactam; CXM: cefuroxime; CRO: ceftriaxone; CPS: cefoperazone-sulbactam; FEP: cefepime; MEM: meropenem; IPM: imipenem; AN: amikacin; GM: gentamicin; CIP: ciprofloxacin; SXT: cotrimoxazole; TGC: tigecycline

Bacteria	AMC	TZP	CXM	CRO	CPS	FEP	MEM	IPM	AN	GM	CIP	SXT	TGC
Escherichia coli (n = 18), n (%)													
Sensitive	6 (33%)	5 (27%)	3 (17%)	2 (11%)	6 (33%)	6 (33%)	10 (56%)	10 (56%)	10 (56%)	10 (56%)	2 (11%)	3 (17%)	15 (83%)
Intermediate	3 (17%)	3 (17%)	5 (27%)	6 (33%)	3 (17%)	0	6 (33%)	5 (27%)	3 (17%)	2 (11%)	6 (33%)	5 (27%)	3 (17%)
Resistant	9 (50%)	10 (56%)	10 (56%)	10 (56%)	9 (50%)	12 (67%)	2 (11%)	3 (17%)	5 (27%)	6 (33%)	10 (56%)	10 (56%)	0
Klebsiella pneumoniae (n = 84), n (%)													
Sensitive	22 (26%)	28 (33%)	21 (25%)	20 (24%)	20 (24%)	14 (16%)	28 (33%)	20 (24%)	14 (16%)	20 (24%)	20 (24%)	14 (16%)	64 (76%)
Intermediate	20 (24%)	6 (7%)	0	14 (16%)	14 (16%)	20 (24%)	0	8 (9%)	20 (24%)	8 (9%)	0	20 (24%)	0
Resistant	42 (50%)	50 (60%)	63 (75%)	50 (60%)	50 (60%)	50 (60%)	56 (67%)	56 (67%)	50 (60%)	56 (67%)	64 (76%)	50 (60%)	20 (24%)
Enterobacter cloacae complex (n = 12), n (%)													
Sensitive	0	6 (50%)	0	6 (50%)	7 (59%)	8 (66%)	10 (83%)	7 (59%)	10 (83%)	11 (92%)	7 (59%)	10 (83%)	11 (92%)
Intermediate	2 (17%)	0	1 (8%)	0	2 (17%)	0	0	2 (17%)	0	0	2 (17%)	0	0
Resistant	10 (83%)	6 (50%)	11 (92%)	6 (50%)	3 (24%)	4 (34%)	2 (17%)	3 (24%)	2 (17%)	1 (8%)	3 (24%)	2 (17%)	1 (8%)
Proteus mirabilis (n = 4), n (%)													
Sensitive	0	3 (75%)	0	0	0	1 (25%)	1 (25%)	0	0	1 (25%)	0	1 (25%)	2 (50%)
Intermediate	1 (25%)	0	1 (25%)	1 (25%)	1 (25%)	0	0	1 (25%)	0	1 (25%)	0	0	0
Resistant	3 (75%)	1 (25%)	3 (75%)	3 (75%)	3 (75%)	3 (75%)	3 (75%)	3 (75%)	4 (100%)	2 (50%)	4 (100%)	3 (75%)	2 (50%)
Morganella morgagnii (n = 2), n (%)													
Sensitive	0	1 (50%)	0	1 (50%)	2 (100%)	0	1 (50%)	0	2 (100%)	1 (50%)	0	0	0
Intermediate	1 (50%)	1 (50%)	0	1 (50%)	0	1 (50%)	0	0	0	1 (50%)	0	0	0
Resistant	1 (50%)	0	2 (100%)	0	0	1 (50%)	1 (50%)	2 (100%)	0	0	2 (100%)	2 (100%)	2 (100%)
Providentia stuartii (n = 2), n (%)													
Sensitive	0	2 (100%)	0	0	0	0	1 (50%)	0	0	0	1 (50%)	1 (50%)	1 (50%)
Intermediate	1 (50%)	0	1 (50%)	0	1 (50%)	0	0	1 (50%)	0	1 (50%)	0	0	0
Resistant	1 (50%)	0	1 (50%)	2 (100%)	1 (50%)	2 (100%)	1 (50%)	1 (50%)	2 (100%)	1 (50%)	1 (50%)	1 (50%)	1 (50%)

**Table 4:** Antimicrobial susceptibility test findings of non-fermenters (n = 78) TZP: piperacillin-tazobactam; CRO: ceftriaxone; CPS: cefoperazone-sulbactam; FEP: cefepime; AN: amikacin; GM: gentamicin; MEM: meropenem; IPM: imipenem; SXT: cotrimoxazole; LVX: levofloxacin; CAZ: ceftazidime; MI: minocycline; ATM: aztreonam; TGC: tigecycline; NA: not applicable

Bacteria	TZP	CRO	CPS	FEP	AN	GM	MEM	IPM	SXT	LVX	CAZ	MI	ATM	TGC
Acinetobacter baumannii (n = 30), n (%)														
Sensitive	3 (10%)	3 (10%)	8 (27%)	3 (10%)	9 (30%)	9 (30%)	8 (27%)	9 (30%)	10 (33%)	3 (10%)	3 (10%)	8 (27%)	NA	21 (70%)
Intermediate	3 (10%)	9 (30%)	0	0	3 (10%)	0	0	0	0	9 (30%)	9 (30%)	8 (27%)		9 (30%)
Resistant	24 (80%)	18 (60%)	22 (73%)	27 (90%)	18 (60%)	21 (70%)	22 (73%)	21 (70%)	20 (67%)	18 (60%)	18 (60%)	14 (46%)		0
Pseudomonas aeruginosa (n = 43), n (%)														
Sensitive	22 (51%)	NA	27 (63%)	22 (51%)	28 (65%)	19 (44%)	22 (51%)	28 (65%)	NA	27 (63%)	22 (51%)	NA	19 (44%)	0
Intermediate	2 (5%)		0	2 (5%)	0	8 (19%)	2 (5%)	0		0	2 (5%)		8 (19%)	0
Resistant	19 (44%)		16 (37%)	19 (44%)	15 (35%)	16 (37%)	19 (44%)	15 (35%)		16 (37%)	19 (44%)		16 (37%)	43 (100%)
Burkholderia cepacia (n = 3), n (%)														
Sensitive	0	0	2 (67%)	0	0	0	2 (67%)	1 (33%)	2 (67%)	2 (67%)	2 (67%)	2 (67%)	0	0
Intermediate	1 (33%)	0	0	1 (33%)	1 (33%)	1 (33%)	0	0	0	0	0	0	1 (33%)	1 (33%)
Resistant	2 (67%)	3 (100%)	1 (33%)	2 (67%)	2 (67%)	2 (67%)	1 (33%)	2 (67%)	1 (33%)	1 (33%)	1 (33%)	1 (33%)	2 (67%)	2 (67%)
Elizabethkingia meningoseptica (n = 2), n (%)														
Sensitive	0	0	0	0	0	0	0	0	0	0	0	1 (50%)	0	NA
Intermediate	0	1 (50%)	1 (50%)	0	0	1 (50%)	0	1 (50%)	0	1 (50%)	1 (50%)	0	1 (50%)	
Resistant	2 (100%)	1 (50%)	1 (50%)	2 (100%)	2 (100%)	1 (50%)	2 (100%)	1 (50%)	2 (100%)	1 (50%)	1 (50%)	1 (50%)	1 (50%)	

**Table 5:** Antimicrobial susceptibility test findings of Gram-positive cocci (n = 22) GM: gentamicin; CIP: ciprofloxacin; SXT: cotrimoxazole; VA: vancomycin; LZD: linezolid; DAP: daptomycin; RA: rifampicin; CM: clindamycin; E: erythromycin; TGC: tigecycline; LVX: levofloxacin; TEC: teicoplanin

Bacteria	GM	CIP	SXT	VA	LZD	DAP	RA	CM	E	TGC	LVX	TEC
Staphylococcus aureus (n = 9), n (%)												
Sensitive	6 (67%)	5 (56%)	6 (67%)	9 (100%)	9 (100%)	9 (100%)	5 (56%)	2 (22%)	0	9 (100%)	5 (56%)	9 (100%)
Intermediate	0	2 (22%)	0	0	0	0	4 (44%)	2 (22%)	3 (33%)	0	2 (22%)	0
Resistant	3 (33%)	2 (22%)	3 (33%)	0	0	0	0	5 (56%)	6 (67%)	0	2 (22%)	0
Enterococcus faecalis (n = 3), n (%)												
Sensitive	0	0	0	2 (67%)	2 (67%)	2 (67%)	0	0	0	2 (67%)	0	2 (67%)
Intermediate	0	1 (33%)	0	0	1 (33%)	0	0	0	1 (33%)	0	1 (33%)	1 (33%)
Resistant	3 (100%)	2 (67%)	3 (100%)	1 (33%)	0	1 (33%)	3 (100%)	3 (100%)	2 (67%)	1 (33%)	2 (67%)	0
Enterococcus faecium (n = 3), n (%)												
Sensitive	0	0	1 (33%)	2 (67%)	2 (67%)	2 (67%)	0	1 (33%)	0	2 (67%)	0	2 (67%)
Intermediate	1 (33%)	1 (33%)	0	0	1 (33%)	0	1 (33%)	0	1 (33%)	0	1 (33%)	1 (33%)
Resistant	2 (67%)	2 (67%)	2 (67%)	1 (33%)	0	1 (33%)	2 (67%)	2 (67%)	2 (67%)	1 (33%)	2 (67%)	0
Streptococcus pneumoniae (n = 7), n (%)												
Sensitive	5 (71%)	5 (71%)	5 (71%)	7 (100%)	7 (100%)	7 (100%)	6 (86%)	2 (29%)	0	6 (86%)	5 (71%)	6 (86%)
Intermediate	0	0	0	0	0	0	0	1 (14%)	3 (43%)	0	1 (14%)	0
Resistant	2 (29%)	2 (29%)	2 (29%)	0	0	0	1 (14%)	4 (57%)	4 (57%)	1 (14%)	1 (14%)	1 (14%)

Klebsiella pneumoniae (n = 84) showed the highest sensitivity to tigecycline (64/84, 76%). They showed maximum resistance to ciprofloxacin (64/84, 76%). Escherichia coli (n = 18) also showed maximum sensitivity to tigecycline (15/18, 83%). They were most resistant to cefepime (12/18, 67%). Enterobacter cloacae complex (n = 12) showed maximum sensitivity to gentamicin and tigecycline (11/12, 92%) isolates. Maximum resistance was seen to cefuroxime (11/12, 92%). Proteus mirabilis (n = 4) showed 100% sensitivity to amikacin and gentamicin but 75% resistance to piperacillin-tazobactam. Morganella morgagnii (n = 2) showed 100% resistance to cefoperazone-sulbactam and amikacin.

Among the non-fermenters isolated, Pseudomonas aeruginosa (n = 43) was the most common one. All isolates (100%) showed resistance to tigecycline. Pseuomonas aeuroginosa is intrinsically resistant to tigecycline. Of note, 65% (28/43) isolates showed the maximum susceptibility to imipenem. Of the 30 isolates of Acinetobacter baumannii grown, 21/30 (70%) were found to be susceptible to tigecycline. But 24/30 (80%) were resistant to piperacillin-tazobactam. Burkholderia cepacia showed good sensitivity to cefoperazone-sulbactam, meropenem, cotrimoxazole, and ceftazidime of 67%.

Nine of the 22 Gram-positive cocci isolated were identified as Staphylococcus aureus. They showed an astounding 100% sensitivity to vancomycin, linezolid, tigecycline, and teicoplanin, but 6/22 (67%) of these isolates were resistant to erythromycin; 7/22 isolates were identified as Streptococcus pneumoniae. All of them (100%) were susceptible to vancomycin, linezolid, and daptomycin. But 57% of them (4/7) showed resistance to clindamycin and erythromycin.

## Discussion

A large proportion of the morbidity and mortality caused by infectious diseases worldwide is attributable to LRTIs. They account for 6% of consultations with doctors, 4.4% of hospital admissions, and 3% to 5% of adult deaths [20]. The range of pathogens responsible for LRTIs varies considerably across regions and over time [21,22]. This study was undertaken to describe the profile of bacterial pathogens isolated from BAL fluid in patients with LRTI, to evaluate the antimicrobial susceptibility patterns of the isolated bacteria, and to determine the association between demographic characteristics and the presence of LRTI and the identified bacteria.

Of the 390 BAL samples included in our study, growth was seen in 222 samples, corresponding to a growth positivity rate of 56.9%. This is comparable to the culture positivity rates reported in studies by Paleawar et al. [23] and Ramana et al. [24], which were 56% and 52.83%, respectively. The 222 patients who were confirmed to have bacterial LRTIs in our study had a mean age of 53.9 years, consistent with findings from studies by Olambe et al. [25]. Of these, 166 (74.8%) were males, and 56 (25.2%) were females. A total of 81 (36.5%) of them reported a positive history of hypertension, which is in agreement with the results of the large-scale prospective study conducted by Zekavat et al. [26].

Of all the pathogens isolated, the most common was Klebsiella pneumoniae (84/222, 37.8%). This finding is consistent with results reported in a study by Padmaja et al. [27]; 157/222 (70.7%) had a positive history of type 2 diabetes mellitus, which is slightly higher than the prevalence reported in the large-scale study done by Klekotka et al. [28]. Klebsiella pneumoniae showed the highest sensitivity to tigecycline (64/84, 76%) and the greatest resistance to ciprofloxacin (64/84, 76%). Similar patterns were observed in a study done by Panigrahi et al. [29], where the tigecycline sensitivity level was 74.79%, and the ciprofloxacin resistance level was 78.40%. Escherichia coli showed the highest sensitivity to tigecycline (83%) and the greatest resistance to cephalosporins, which was consistent with findings from a study by Mohapatra et al. [29].

Pseudomonas aeruginosa showed high sensitivity to amikacin and imipenem (65%, 28/43). Isolates of Acinetobacter baumannii were highly susceptible to tigecycline (70%) and showed high resistance to piperacillin-tazobactam (80%). Both these organisms showed similar patterns of susceptibility in a study by Choudhury et al. [30]. Staphylococcus aureus showed 100% susceptibility to vancomycin, linezolid, daptomycin, and teicoplanin but a high level of resistance to clindamycin (56%) and erythromycin (67%). These observations are consistent with those reported in the study by Mohapatra et al. [31].

Strengths and limitations

This study included a subgroup analysis based on the type of pathogen isolated (Enterobacterales, non-fermenters, and Gram-positive cocci). However, as a retrospective study, it had missing data, and the results could have been more robust with the inclusion of information on hospital stay, other coexisting infections, and patient outcomes. These constitute the major limitations of the study. A prospective study design and a larger sample size would have strengthened the findings.

## Conclusions

We observed a predominance of Gram-negative bacteria causing LRTIs, particularly Klebsiella pneumoniae. The high culture positivity rate underscores the significance of bronchoalveolar lavage as a tool for the effective diagnosis of LRTIs. Future multicentric, prospective studies that integrate molecular resistance profiling and clinical outcomes are recommended to optimize empirical therapy and thereby enhance antimicrobial stewardship practices.
